# Supplementary material for: Hybrid Models and Biological Model Reduction with PyDSTool
Source: PLoS Comput Biol. 2012 Aug 9;8(8):e1002628. doi: 10.1371/journal.pcbi.1002628 (PMC3415397; doi:10.1371/journal.pcbi.1002628)
Supplement: Text S4 — Complete source code for the PyDSTool package (version 0.88.120504). Includes API documentation and help files linking to web pages. This file is identical to the current public release on Sourceforge.net. (ZIP) [file pcbi.1002628.s004.zip › PyDSTool/html/PyDSTool.Toolbox.optimizers.line_search.adaptive_last_step_modifier-pysrc.html]

xml version="1.0" encoding="ascii"?


PyDSTool.Toolbox.optimizers.line\_search.adaptive\_last\_step\_modifier


| Home | Trees | Indices | Help | PyDSTool |
| --- | --- | --- | --- | --- |

Package PyDSTool ::
Package Toolbox ::
Package optimizers ::
Package line\_search ::
Module adaptive\_last\_step\_modifier

[hide private]

[frames] | no frames]

# Source Code for Module PyDSTool.Toolbox.optimizers.line\_search.adaptive\_last\_step\_modifier

```
 1   
 2  # Matthieu Brucher 
 3  # Last Change : 2007-08-28 00:34 
 4   
 5  """ 
 6  Line search decorator that overrides the default alpha_step value with a factor times the last alpha_step, the factor being a function of the current direction and the last direction 
 7  """ 
 8   
 9  import numpy 
10   


11 -class AdaptiveLastStepModifier(object):


12    """ 
13    Overrides the default step size and replaces it with a factor times the last one 
14    """ 


15 -  def __init__(self, line_search, **kwargs):


16      """ 
17      Needs to have : 
18        - the decorated line search (line_search) 
19      """ 
20      self.line_search = line_search

21   


22 -  def __call__(self, origin, function, state, **kwargs):


23      """ 
24      Returns a good candidate 
25      Parameters : 
26        - origin is the origin of the search 
27        - function is the function to minimize 
28        - state is the state of the optimizer 
29      """ 
30      if 'alpha_step' in state: # in this case, last_direction and last_gradient should be defined 
31        state['initial_alpha_step'] = numpy.dot(state['last_gradient'], state['last_direction']) / numpy.dot(state['gradient'], state['direction']) * state['alpha_step'] 
32      state['last_direction'] = state['direction'] 
33      state['last_gradient'] = state['gradient'] 
34      return self.line_search(origin = origin, function = function, state = state, **kwargs)

35
```

  


| Home | Trees | Indices | Help | PyDSTool |
| --- | --- | --- | --- | --- |

|  |  |
| --- | --- |
| Generated by Epydoc 3.0.1 on Fri May 4 15:24:14 2012 | http://epydoc.sourceforge.net |
